# Single-Cell Transcriptomic Landscape of Right-Sided Colon Cancer Reveals Cellular and Molecular Features of Metastatic Potential

**DOI:** 10.3390/biomedicines14030563

**Published:** 2026-02-28

**Authors:** Zhixin Ye, Wanrui Zhang, Hongshen Qiu, Feng Luo, Changyi Liao, Kai Lei, Qi Zhou

**Affiliations:** 1 Department of Obstetrics and Gynecology, The First Affiliated Hospital, Sun Yat-sen University, Guangzhou 510080, China; 2 Center of Translational Medicine, The First Affiliated Hospital, Sun Yat-sen University, Guangzhou 510080, China; 3 Center of Pulmonary and Critical Care Medicine, The First Affiliated Hospital, Sun Yat-sen University, Guangzhou 510080, China; 4 Department of Radiation Oncology, The First Affiliated Hospital of Guangzhou Medical University, Guangzhou 510120, China; 5 Department of General Surgery, Hui Ya Hospital of The First Affiliated Hospital, Sun Yat-sen University, Huizhou 516081, China; 6 Department of Hepatobiliary Surgery, Hui Ya Hospital of The First Affiliated Hospital, Sun Yat-sen University, Huizhou 516081, China; 7 Center of Hepato-Pancreato-Biliary Surgery, The First Affiliated Hospital, Sun Yat-sen University, Guangzhou 510080, China

**Keywords:** right-sided colon cancer, liver metastasis, single-cell RNA sequencing, metabolic reprogramming

## Abstract

**Background**: Right-sided colon cancer (RCC) is clinically aggressive and prone to liver metastasis, yet the cellular basis underlying its metastatic potential remains unclear. This study aimed to delineate the single-cell landscape of primary RCC tumors with and without liver metastasis. **Methods**: Public single-cell RNA sequencing datasets of primary right-sided colon tumors from eight patients (five with liver metastasis and three without metastasis) were integrated and analyzed. Malignant cells were identified by copy number variation inference. Tumor subclusters, differential gene expression, pathway enrichment, metabolic activity, and pseudotime trajectories were systematically compared between RCC with liver metastasis (RCC_LM) and without metastasis (RCC_noM). **Results**: RCC_LM tumors exhibited higher genomic instability and a significantly higher proportion of cells in G1 phase, suggesting that altered cell cycle progression is a key feature of tumors with metastatic potential. Five tumor subclusters were identified, with stem-like tumor cells significantly enriched in RCC_LM, whereas enterocyte-like cells predominated in RCC_noM. The primary tumor samples from tumors that metastasized displayed transcriptional programs indicative of epithelial–mesenchymal transition, extracellular matrix remodeling, inflammatory signaling, and metabolic reprogramming involving glycolysis and oxidative phosphorylation. Trajectory analyses indicated that RCC_LM tumors were enriched in early pseudotime states, suggesting increased cellular plasticity. **Conclusions**: These findings indicate that liver metastatic potential in RCC is marked by stem-like tumor states, metabolic plasticity, and microenvironmental remodeling, providing insight into the cellular mechanisms underlying RCC progression.

## 1. Introduction

Colorectal cancer (CRC) is the third most common malignancy and the second leading cause of cancer-related death worldwide [1]. A major challenge in CRC management is distant metastasis, particularly to the liver, which affects up to 50% of patients and significantly worsens prognosis [2]. Despite advances in surgical techniques and systemic therapies, the molecular and cellular mechanisms driving liver metastasis in CRC remain incompletely understood.

Right-sided colon cancer (RCC), which arises from the cecum, ascending colon, and hepatic flexure, differs markedly from left-sided tumors in terms of epidemiology, clinical behavior, and molecular features [3,4]. Right-sided colon cancer (RCC) is often associated with microsatellite instability (MSI-H), the CpG island methylator phenotype (CIMP), and a distinct immune microenvironment [5,6]. Epidemiological studies have consistently shown that MSI-H tumors are more frequently located in the right colon; for instance, a Brazilian cohort demonstrated that MSI-positive colorectal cancers predominantly arise on the right side [7], and multiple studies have confirmed a higher prevalence of MSI-H and CIMP-high phenotypes in right-sided tumors [8]. Historically, MSI-H colorectal cancers were considered to have a poorer prognosis in the era of conventional chemotherapy, largely due to resistance to 5-fluorouracil-based adjuvant treatment [6]. However, the advent of immune checkpoint inhibitors (ICIs) has fundamentally transformed the outlook for these patients. MSI-H is now recognized as a powerful predictive biomarker for ICI efficacy: the U.S. Food and Drug Administration (FDA) has approved pembrolizumab as first-line therapy for metastatic MSI-H colorectal cancer [9], and clinical trials have reported objective response rates of 40–65% in MSI-H tumors treated with PD-1 blockade [10,11]. Consequently, the high frequency of MSI-H in RCC, rather than contributing to a uniformly poor prognosis, now identifies a subgroup that is particularly likely to benefit from immunotherapy, underscoring the importance of molecular subtyping in guiding treatment decisions.

The clinical relevance of liver metastasis in RCC is underscored by distinct epidemiological and prognostic features. Patients with right-sided colon cancer exhibit a higher propensity for synchronous liver metastasis, with studies demonstrating that among colorectal cancer patients presenting with synchronous metastases, RCC accounts for a significantly higher proportion than left-sided colon cancer (LCC) [12]. Furthermore, RCC patients with synchronous metastasis more frequently display lymphovascular invasion and liver involvement compared to those with metachronous metastasis [12]. In the metastatic setting, the prognostic disadvantage of RCC becomes particularly pronounced. Patients with RCC-derived colorectal liver metastases (CRLM) have significantly worse outcomes than their LCC counterparts. Among patients with metastatic disease receiving anti-EGFR therapy, the RCC group demonstrated markedly inferior median overall survival (10.1 months), progression-free survival, and objective response rate compared to the LCC group [13]. This survival disadvantage persists even after stratifying by KRAS mutation status, with some studies suggesting that right-sided primary tumors independently predict poorer outcomes in patients with CRLM [14].These clinical differences likely stem from underlying biological heterogeneity. Emerging evidence indicates that RCC and LCC liver metastases exhibit distinct metabolic profiles, with multi-omics analyses revealing significant differences in tumor cell metabolism that may constitute the molecular basis for their divergent clinical behavior [15]. However, the specific cellular and transcriptional alterations within RCC that predispose to liver metastasis remain poorly characterized.

Previous studies using bulk transcriptomics, genomics, and peripheral blood biomarkers have provided valuable insights into the metastatic cascade of CRC [16,17]. Nevertheless, bulk approaches obscure the heterogeneity of malignant cells and fail to resolve the diverse stromal and immune components of the tumor microenvironment (TME) [18]. This limitation hampers our understanding of how cellular subpopulations cooperate or compete to enable metastatic dissemination. Systematic investigations of the cellular architecture and dynamic trajectories of RCC tumors, especially those with or without liver metastasis, at single-cell resolution are scarce.

Single-cell RNA sequencing (scRNA-seq) has revolutionized cancer research by enabling unbiased profiling of thousands of individual cells. This approach captures transcriptional heterogeneity and reveals rare subpopulations that may drive disease progression [19,20]. Moreover, trajectory inference methods, such as Monocle, allow for the reconstruction of cell state transitions, offering insights into the dynamic processes underlying tumor evolution and metastatic potential. Applying these techniques to CRC, and specifically to RCC, holds promise for identifying novel biomarkers and therapeutic targets for metastasis prevention.

In this study, we collected and analyzed scRNA-seq data from right-sided colon tumors of patients with and without liver metastasis. Through integrated analysis, visualization, differential expression profiling, and trajectory inference, we systematically compared the cellular composition, transcriptional programs, and lineage dynamics of the two groups. Our study provides a comprehensive single-cell atlas of RCC in the context of metastatic progression and uncovers candidate cellular and molecular drivers of liver metastasis. These findings advance our understanding of RCC biology and may inform strategies for risk stratification and therapeutic intervention in metastatic disease.

## 2. Methods

### 2.1. scRNA-Seq Data Preprocessing

Raw scRNA-seq data were collected from two public datasets (GSE245552 and GSE188711), focusing on tumor samples originating from the right-sided colon [21,22]. A total of eight cases were retained for analysis (five from GSE245552 and three from GSE188711). Dataset GSE245552 contains primary tumor samples from 18 colorectal cancer patients. Among these, five were identified as right-sided colon tumors based on anatomical site annotations provided in the original publication and supplementary clinical information (https://www.ncbi.nlm.nih.gov/geo/query/acc.cgi?acc=GSE245552, accessed on 25 February 2026). These samples correspond to the following GEO accession numbers: GSM7844812 (Pt01_T1), GSM7844823 (Pt06_T1), GSM7844833 (Pt10_T1), GSM7844837 (Pt13_T1), and GSM7844844 (Pt17_T1). According to the source study, all five patients presented with synchronous liver metastasis at the time of primary tumor resection; thus, these samples were assigned to the liver metastasis group (RCC_LM). Dataset GSE188711 includes six primary colorectal cancer samples, of which three are explicitly annotated as right-sided colon tumors. These samples correspond to the following GEO accession numbers: GSM5688709 (Right-sided CRC 1), GSM5688710 (Right-sided CRC 2), and GSM5688711 (Right-sided CRC 3). All three were confirmed as non-metastatic (M0) based on clinical metadata and were included in the non-metastatic group (RCC_noM). A total of eight right-sided colon cancer samples (five from GSE245552 and three from GSE188711) were retained for downstream analysis. Both datasets were generated using droplet-based 10x Genomics technologies, enabling high-throughput single-cell transcriptome capture and sequencing. Raw count matrices for the two datasets were obtained from GEO as pre-processed by the original studies using the Cell Ranger pipeline (version 5.0.1 for GSE245552, version 3.0 for GSE188711) aligned to the human reference genome hg38 (GRCh38). Gene symbols in both datasets were originally derived from Ensembl annotation. After merging the datasets using the merge function in Seurat (which takes the union of all genes), a total of 33,583 unique gene symbols were detected. This count includes both protein-coding genes and non-coding RNAs, consistent with the complexity of the human transcriptome. To ensure consistency with current official nomenclature, we cross-referenced all gene symbols against the HUGO Gene Nomenclature Committee (HGNC) approved list (retrieved via biomaRt, comprising 41,365 unique symbols). Genes that did not directly match were processed using the HGNChelper R package to update outdated aliases. This resulted in a final set of 20389 HGNC-approved genes for all downstream analyses. Gene expression count matrices based on unique molecular identifiers (UMIs) were imported into the R environment (v4.3.1) and processed using the Seurat package (v4.3.0.1) [23]. Low-quality cells were excluded if they expressed fewer than 300 genes, contained more than 15% mitochondrial transcripts, or exceeded 10,000 total UMI counts. These thresholds were chosen based on the distribution of these metrics across the dataset to exclude potential doublets, empty droplets, and dying cells while retaining high-quality singlets. To minimize artifacts from doublets, the DoubletFinder algorithm (v2.0.3) was applied with an expected doublet rate of 7.5%, optimized using pN = 0.25 (artificial doublet proportion) and pK (neighborhood size for pANN calculation) via Bayesian Information Criterion [24]. Only high-confidence singlets were retained, resulting in an integrated dataset of 31,674 high-quality single cells across 20,389 expressed genes for subsequent analyses.

### 2.2. Single Cell, Tumor Cell Dimensionality Reduction, Clustering, and Annotation

**Figure 1:**
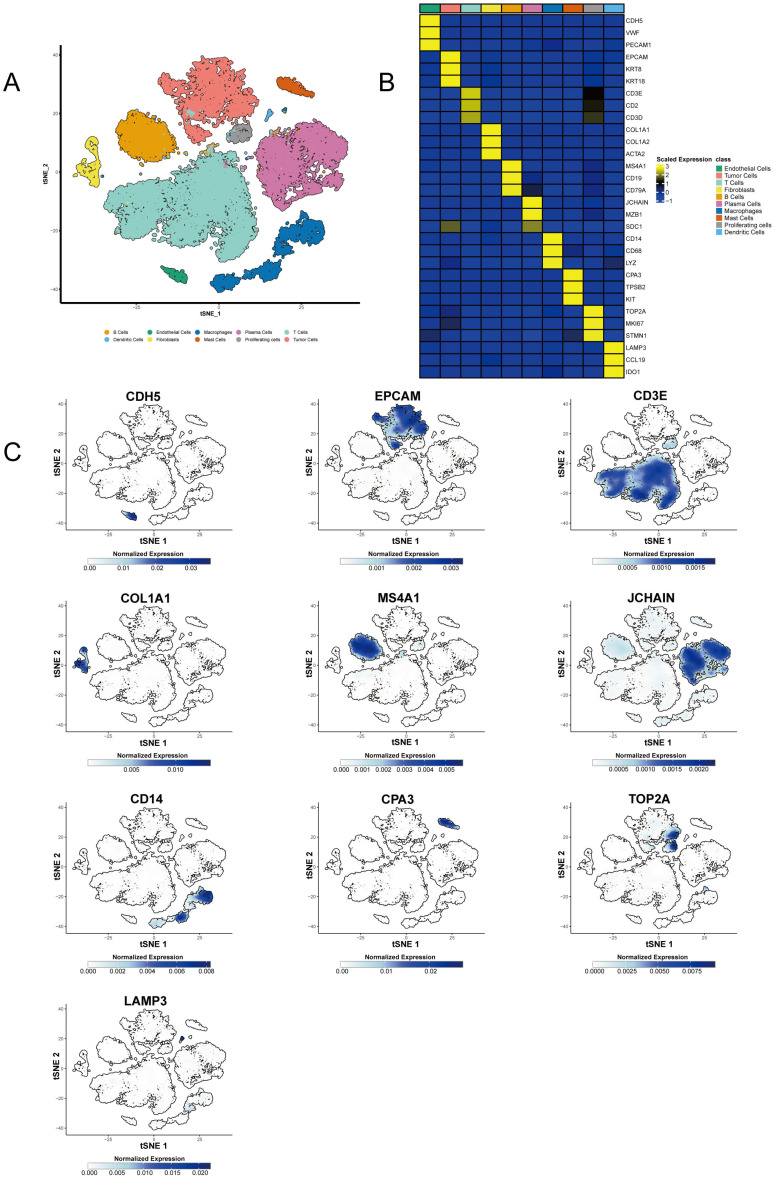
t-SNE analysis and differential gene expression in the tumor microenvironment of right-sided colon cancer. (**A**) t-SNE plot showing the clustering of different cell types within the right-sided colon tumor microenvironment. The cell types, including T cells, B cells, plasma cells, macrophages, endothelial cells, fibroblasts, mast cells, proliferating cells, dendritic cells, and tumor cells, are color-coded based on their expression of lineage-specific markers. (**B**) Heatmap of differential expression of key cell markers, including *CD3E*, *CD2*, and *CD3D* (T cells), *MS4A1*, *CD19*, and *CD79A* (B cells), *JCHAIN*, *MZB1*, and *SDC1* (plasma cells), *CD14*, *CD68*, and *LYZ* (macrophages), *COL1A1*, *COL1A2*, and *ACTA2* (fibroblasts), *CDH5*, *VWF*, and *PECAM1* (endothelial cells), *EPCAM*, *KRT18*, and *KRT8* (tumor cells), *CPA3*, *TPSB2*, and *KIT* (mast cells), *TOP2A*, *MKI67*, and *STMN1* (proliferating cells), and *LAMP3*, *CCL19*, and *IDO1* (dendritic cells). Expression levels are color-coded from low (blue) to high (yellow). Colors in both (**A**) and the top annotation of (**B**) have been selected to be distinguishable for color-blind readers (Okabe–Ito palette). (**C**) t-SNE plots showing the expression of key markers (*CD3E*, *CD2*, *CD3D*, *MS4A1*, *CD19*, *CD79A*, *JCHAIN*, *MZB1*, *CD14*, *CD68*, *LYZ*, *COL1A1*, *COL1A2*, *ACTA2*, *CDH5*, *VWF*, *PECAM1*, *EPCAM*, *KRT18*, *KRT8*, *CPA3*, *TPSB2*, *KIT*, *TOP2A*, *MKI67*, *STMN1*, *LAMP3*, *CCL19*, *IDO1*, etc.) across the tumor microenvironment. Gene expression intensity is color-coded from low (blue) to high (dark blue), illustrating the spatial distribution of these markers within the t-SNE clusters.

**Figure 2:**
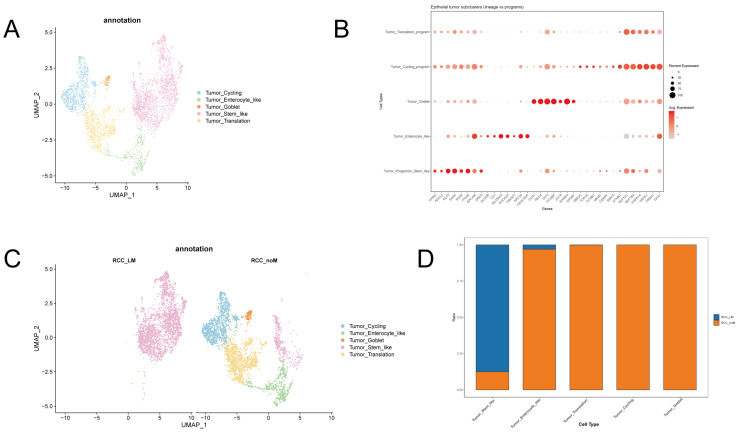
Tumor subcluster identification and proportions in right-sided colon cancer. (**A**) UMAP plot showing five tumor subclusters, identified based on gene expression profiles. Subclusters are annotated as Tumor Stem-like, Tumor Translation-high, Tumor Cycling, Tumor Enterocyte-like, and Tumor Goblet/Secretory. (**B**) Dot plot displaying the expression of key marker genes across each tumor subcluster, with dot size reflecting the percentage of cells expressing the marker and color indicating the average expression level. (**C**) UMAP plot showing the distribution of tumor subclusters in the RCC_LM (liver metastasis) and RCC_noM (no metastasis) groups. (**D**) Bar plot showing the proportion of each tumor subcluster in RCC_LM and RCC_noM groups, highlighting a higher prevalence of Tumor Stem-like cells in RCC_LM and Tumor Enterocyte-like cells in RCC_noM.

For both the integrated single-cell dataset and the subset of tumor cells, dimensionality reduction and clustering analyses were performed. After log-normalization (NormalizeData) and identification of the top 2000 highly variable features (FindVariableFeatures), data scaling (ScaleData) was applied; technical covariates such as nCount_RNA and percent.mt were not explicitly regressed out, as we aimed to preserve potential biological signals related to mitochondrial function and metabolic activity—low-quality cells having been removed during initial quality control (Section 2.1). Principal component analysis (PCA) was performed on the scaled data, and both an ElbowPlot and JackStraw analysis were used to determine the number of principal components (PCs) for downstream analysis; based on these evaluations, the top 32 principal components (PCs) were selected as they captured the majority of the biological variance while excluding noise, as determined by ElbowPlot and JackStraw analyses. To correct for technical variations between the two source datasets (GSE188711 and GSE245552), the Harmony algorithm (v1.2.0) was applied to these 32 PCs [25]. This algorithm iteratively clusters the data to maximize diversity between batches, effectively aligning cells from different samples based on their biological similarity. The corrected Harmony embeddings were subsequently employed for dimensionality reduction with method-specific applications: for global single-cell atlas visualization (Figure 1 and Results Section 3.1), t-SNE was performed (RunTSNE, dims = 1:32) to resolve major cell lineages; for refined subclustering analysis of malignant cells (Figure 2 and Results Section 3.2), UMAP was applied (RunUMAP, dims = 1:25) to enhance separation of biologically subtle subpopulations. Graph-based clustering was executed directly on the Harmony embeddings using FindClusters (Louvain algorithm), independent of visualization method. The resolution parameter was systematically optimized: resolution = 0.1 for major lineage identification (yielding T cells, B cells, fibroblasts, etc., validated by canonical markers); resolution = 0.1 for malignant cell subclustering (generating five biologically coherent subclusters without over-splitting). All clusters were validated via marker gene expression, and visualizations were explicitly labeled per analytical purpose (t-SNE for Figure 1; UMAP for Figure 2).

### 2.3. Copy Number Variation (CNV) Inference with InferCNV

To delineate malignant epithelial populations and confirm large-scale genomic alterations, we applied both inferCNV (v1.16.0, https://github.com/broadinstitute/inferCNV, accessed on 25 February 2026). The analysis was performed using B cells as a diploid reference population. Raw UMI count matrices were preprocessed with a gene expression cutoff of 0.1, denoising enabled (denoise = TRUE), and the hidden Markov model turned off (HMM = FALSE), following the recommended settings for CNV inference at the single-cell level. This approach provided estimates of broad chromosomal copy number shifts, allowing us to detect large-scale genomic alterations in the tumor microenvironment.

For further quantitative analysis, we calculated a CNV score for each cell. This score was computed as the mean of squared deviations of gene expression values from the baseline expression level of 1 (as estimated by inferCNV relative to the reference B cells). A higher CNV score indicates greater large-scale chromosomal copy number alterations and serves as a proxy for the degree of genomic instability within a cell. To assess the potential influence of sex chromosomes on CNV inference, we performed a sensitivity analysis by repeating the entire inferCNV procedure using only autosomal genes. Genes located on chromosomes X and Y were excluded from the expression matrix based on the gene order file. CNV scores were recalculated as described above and compared with those obtained from the full gene set. The CNV scores were then associated with specific cell groups, including B cells, Proliferating cells, and Tumor cells, to investigate group-level differences in CNV alterations.

Unsupervised clustering was employed to identify distinct cell populations based on CNV scores. Statistical testing, including Kruskal–Wallis and Wilcoxon tests, was performed to assess the differences in CNV scores between these groups. Significant differences between groups were visualized using violin plots with overlaid boxplots, and statistical significance was indicated with *p*-values derived from the Kruskal–Wallis test and pairwise comparisons (adjusted by the Benjamini–Hochberg method).

In addition to group-level comparisons, we performed CNV analysis across different subtypes of colon cancer, specifically comparing RCC_LM and RCC_noM groups based on the dataset source. The CNV scores were compared using Kruskal–Wallis tests followed by pairwise Wilcoxon tests to identify potential differences in CNV profiles between these two groups.

### 2.4. Trajectory, Pseudotime, and Differentiation Potential Analysis with Monocle2 and CytoTRACE

To explore dynamic state transitions of malignant epithelial cells, we performed trajectory analysis using Monocle2 and assessed differentiation potential using CytoTRACE [26,27].

For Monocle2 analysis, Seurat objects containing malignant epithelial cells were converted into Monocle2 CellDataSets. Normalization was performed by estimating size factors and dispersions, and highly variable genes were selected as ordering features. Dimensionality reduction was carried out using the DDRTree algorithm, followed by pseudotime ordering with the orderCells function. In some analyses, the root state was defined according to transcriptional signatures characteristic of more differentiated epithelial populations. Branch-dependent gene expression dynamics were then tested using BEAM and visualized along the pseudotime trajectory, enabling identification of lineage-specific programs associated with metastatic potential.

To further assess the differentiation potential of malignant epithelial cells, we applied CytoTRACE (version 0.3.3). This method predicts cellular differentiation states based on the negative correlation between transcriptional diversity (the number of expressed genes per cell) and developmental potential. Raw count matrices were extracted from the epithelial subset, and genes detected in fewer than five cells were excluded to reduce technical noise. The CytoTRACE function was executed with default parameters to generate a differentiation score for each cell, with higher scores indicating greater stemness and lower differentiation.

### 2.5. Differential Expression and Pathway Enrichment

Differential expression was assessed within the epithelial subset by comparing cells from RCC with liver metastasis (RCC_LM) and RCC without metastasis (RCC_noM), as defined by the dataset_source metadata. The Wilcoxon rank-sum test implemented in Seurat (FindMarkers, min.pct = 0.25, logfc.threshold = 0) was used, with multiple testing correction by the Benjamini–Hochberg (BH) procedure. Genes were ranked by log2 fold-change for subsequent enrichment analyses. Gene sets were retrieved from MSigDB, including HALLMARK and REACTOME collections via the msigdbr package [28]. Enrichment was performed using fgseaMultilevel, with parameters set to minSize = 10 and maxSize = 500. Normalized enrichment scores (NES) and adjusted *p*-values (padj) were used to identify significantly enriched pathways (padj < 0.05). Pathways with positive NES were considered enriched in RCC_LM, while those with negative NES were considered enriched in RCC_noM. Notably, all enrichment results were derived from ranked gene lists.

### 2.6. Metabolic Pathway Activity Analysis

Metabolic activity at the pathway level was quantified using a weighted relative pathway activity algorithm originally developed by Xiao et al. [29] for single-cell metabolic analysis. This method is based on KEGG metabolism gene sets [30] and follows the established computational framework.

For each cell type, the mean expression of metabolic genes was calculated and normalized against their global average across all cells to obtain relative expression values. For each pathway, a weighted mean of the relative expression values was computed, where weights were defined as the inverse of the number of pathways in which each gene appeared. This weighting strategy reduces the contribution of genes that are pleiotropic and participate in multiple metabolic pathways [29].

To minimize the influence of low-abundance or highly variable genes, outliers were excluded if their relative expression exceeded three times the 75th percentile or fell below one-third of the 25th percentile within a given pathway, following the conservative criteria established in the original method. Statistical significance of pathway activity scores was assessed by a permutation test with 5000 random shuffles of cell labels, generating an empirical null distribution. *p*-values were computed by comparing observed scores with the permuted background, and non-significant scores were masked in downstream visualization. Final heatmaps were annotated by functional categories, enabling direct comparison of RCC_LM versus RCC_noM metabolic programs.

### 2.7. Statistical Analysis

All statistical analyses were conducted in the R environment (v4.3.1). To ensure deterministic and reproducible results, the random seed was set at the beginning of each analysis session using set.seed(1). Unless otherwise specified, default parameters of the corresponding R packages were used. For single-cell RNA-seq preprocessing, cell- and gene-level quality control filters were applied as described above. Differential gene expression between RCC with liver metastasis (RCC_LM) and RCC without metastasis (RCC_noM) was tested using the Wilcoxon rank-sum test implemented in the Seurat FindMarkers function, with *p*-values adjusted for multiple testing by the Benjamini–Hochberg (BH) procedure. Genes with adjusted *p*-values (padj < 0.05) were considered statistically significant. For pathway-level analyses, gene set enrichment analysis (GSEA) was performed using fgseaMultilevel [31], and significance was determined by adjusted *p*-values < 0.05. Metabolic pathway activity scores were compared across cell groups by a permutation-based test with 5000 iterations, and empirical *p*-values < 0.05 were regarded as significant. All statistical results, including CNV inference, pseudotime ordering, and enrichment analyses, were interpreted in the context of multiple lines of evidence, and non-significant results were reported as descriptive observations without over-interpretation.

## 3. Results

### 3.1. Cellular Composition and Marker Identification of the Right-Sided Colon Cancer Tumor Microenvironment

We obtained raw single-cell RNA sequencing (scRNA-seq) data from two publicly available datasets (GSE245552 and GSE188711), focusing on tumor samples originating from the right-sided colon. A total of eight cases were retained for analysis (five from GSE245552 and three from GSE188711). To explore the cellular composition of the tumor microenvironment, we performed t-SNE for dimensionality reduction, which revealed distinct cell clusters (Figure 1A). Unsupervised clustering identified several major cell populations, including tumor cells, endothelial cells, fibroblasts, macrophages, B cells, T cells, mast cells, and others.

To characterize these populations, we performed differential gene expression analysis. T cells were primarily marked by *CD3E*, *CD2*, and *CD3D* (Figure 1B,C and Figure S1C). B cells and plasma cells were defined by *MS4A1*, *CD19*, and *CD79A*, and JCHAIN, MZB1, and SDC1, respectively (Figure 1B,C and Figure S1E,F). Macrophages were identified by *CD14*, *CD68*, and *LYZ* (Figure 1B,C and Figure S1G), while fibroblasts were marked by *COL1A1*, *COL1A2*, and *ACTA2* (Figure 1B,C and Figure S1D). Endothelial cells expressed *CDH5*, *VWF*, and *PECAM1* (Figure 1B,C andFigure S1A), and tumor cells exhibited high expression of *EPCAM*, *KRT18*, and *KRT8* (Figure 1B,C and Figure S1B). Mast cells were identified using *CPA3*, *TPSB2*, and *KIT* (Figure 1B,C and Figure S1H). Proliferating cells expressed *TOP2A*, *MKI67*, and *STMN1* (Figure 1B,C and Figure S1I), while dendritic cells were marked by *LAMP3*, *CCL19*, and IDO1 (Figure 1B,C and Figure S1J).

The expression of these markers across the t-SNE plots in Figure 1C and Figure S1 highlights the complexity and cellular heterogeneity of the tumor microenvironment. The distinct expression patterns of markers such as *CD3E*, *MS4A1*, *PECAM1*, *COL1A1*, and others confirm the presence of these cell types and further elucidate their roles in the colon cancer tumor microenvironment.

### 3.2. Tumor Subclusters and Their Proportions in Right-Sided Colon Cancer

**Figure 3:**
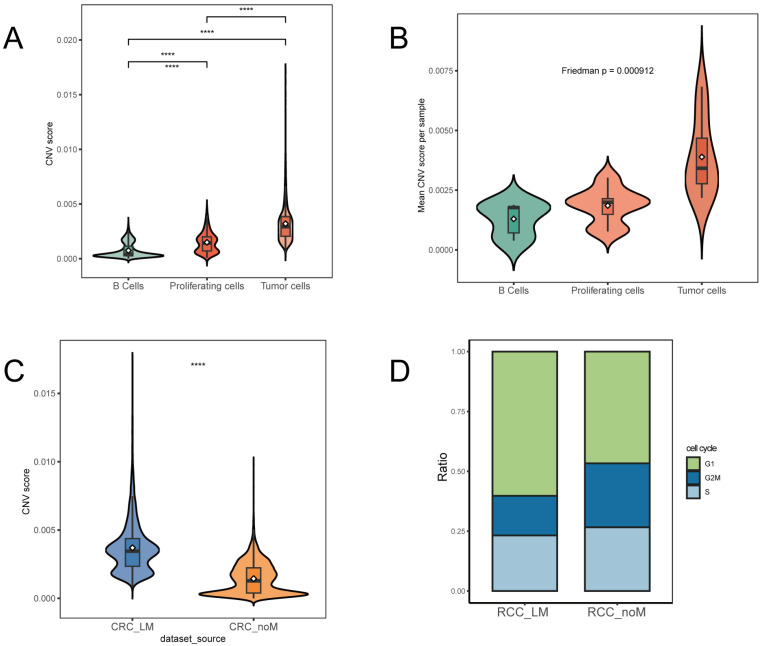
CNV scores and cell cycle distribution in the right-sided colon cancer tumor microenvironment. (**A**) Violin plot comparing CNV scores across B cells, Proliferating cells, and Tumor cells. Tumor cells exhibited significantly higher CNV scores compared to B cells and Proliferating cells (**** *p* < 0.0001, Kruskal–Wallis test and pairwise Wilcoxon test). (**B**) CNV scores across cell types at the patient level. Boxplots show the distribution of mean CNV scores per patient for B cells, proliferating cells, and tumor cells. Each dot represents one patient sample. The Friedman test was used to compare CNV scores among the three cell types (*p* = 0.000912). (**C**) Violin plot comparing CNV scores between the RCC_LM (liver metastasis) and RCC_noM (no metastasis) groups. RCC_LM samples showed significantly higher CNV scores than RCC_noM samples (**** *p* < 0.0001, Kruskal–Wallis test and pairwise Wilcoxon test). (**D**) Stacked bar plot showing the cell cycle distribution of G1, G2M, and S phases in RCC_LM and RCC_noM groups. The RCC_LM group exhibited a higher proportion of cells in the G1 phase, suggesting an altered cell cycle profile consistent with a dormant/migratory state.

We performed dimensionality reduction and clustering to identify distinct subclusters within the tumor cell population of right-sided colon cancer. Five tumor subclusters were identified, including Tumor Stem-like, Tumor Translation-high, Tumor Cycling, Tumor Enterocyte-like, and Tumor Goblet/Secretory (Figure 2A). To address potential confounding effects arising from differences in sequencing platforms and sample processing between the two public datasets (GSE188711 and GSE245552), we performed integrative analysis using the Harmony algorithm. As shown in Figure S2A, after Harmony correction, cells from both datasets are effectively integrated, with tumor cell subtypes forming coherent clusters that span across original sample sources. Notably, the GSE245552 dataset is enriched for Tumor_Stem_like cells, while GSE188711 shows a more diverse tumor cell composition. However, within each biological cluster, cells from different datasets are intermixed, demonstrating successful removal of batch effects. In contrast, without Harmony correction, cells from the two datasets remain segregated into distinct clusters (Figure S2B), confirming the presence of a strong technical batch signal prior to integration. These results validate that our downstream analyses comparing RCC_LM and RCC_noM groups are conducted on biologically aligned data, minimizing the influence of dataset-specific artifacts. The Tumor Stem-like subcluster, characterized by high expression of stem cell-related genes such as *AXIN2*, *ASCL2*, and *KLF5*, was characterized by epithelial markers like *EPCAM* and *ITGA6*, and exhibited epithelial plasticity with markers such as *AREG* and *TNS4* (Figure 2B). The subcluster, which we annotated as ‘Tumor_Translation-high’, was distinguished by a unique gene signature indicating high metabolic and translational activity. Specifically, it showed high expression of key functional markers for protein synthesis and quality control (e.g., EEF1A1, HSPA1A) and a robust oxidative stress response (e.g., PRDX1, GPX1), suggesting a state of elevated protein production tightly coupled with a compensatory antioxidant defense mechanism (Figure 2B). The Tumor Cycling subcluster showed enrichment of cell cycle genes, including *TOP2A*, *CCNB1*, and *STMN1*, characteristic of proliferating cells (Figure 3B). The Tumor Enterocyte-like subcluster exhibited markers such as *GUCA2A/B*, *ALDOB*, and *KRT20*, indicative of intestinal epithelial differentiation (Figure 2B). The Tumor Goblet/Secretory subcluster, marked by *REG4*, *TFF3*, and *MUC1*, was characteristic of goblet cell differentiation (Figure 2B).

Additionally, we analyzed the proportions of these subclusters in the RCC_LM (liver metastasis) and RCC_noM (no metastasis) groups. Tumor Stem-like cells were more prevalent in the RCC_LM group, revealing an enrichment of stem-like characteristics in tumors with metastatic potential. (Figure 2C,D). Conversely, the Tumor Enterocyte-like subcluster was more abundant in RCC_noM, consistent with a more differentiated phenotype in tumors without metastasis (Figure 2C,D).

### 3.3. CNV Score and Cell Cycle Analysis in Right-Sided Colon Cancer

The CNV score analysis (Figure 3A,B) revealed that Tumor cells had significantly higher CNV scores compared to B cells and Proliferating cells, with the difference being highly significant (*p* < 0.0001, Kruskal–Wallis test and pairwise Wilcoxon test). Proliferating cells also exhibited higher CNV scores than B cells, a pattern indicative of genomic instability associated with tumor growth and division. To account for pseudoreplication, CNV scores were aggregated at the patient level by calculating the mean CNV score per cell type for each patient sample. A Friedman test revealed a significant overall difference in CNV scores among B cells, proliferating cells, and tumor cells (*p* = 0.000912; Figure 3B). When comparing the RCC_LM (liver metastasis) and RCC_noM (no metastasis) groups (Figure 3C), RCC_LM tumors showed significantly higher CNV scores, indicating that greater genomic instability correlates with the presence of liver metastasis.

Additionally, cell cycle analysis (Figure 3D) revealed distinct distributions between the RCC_LM and RCC_noM groups. The RCC_LM group exhibited a significantly higher proportion of cells in the G1 phase and a lower proportion in the S phase compared to the RCC_noM group. This pattern is consistent with altered cell cycle progression in metastatic tumors, which may involve G1-phase arrest or a shift toward a dormant/migratory state, though functional studies are needed to confirm these possibilities. Together, these observations reveal an association between cell cycle regulation is dysregulated in metastatic-prone tumors, which may contribute to their aggressive behavior through mechanisms beyond rapid proliferation.

### 3.4. Differential Gene Expression and Metabolic Pathway Activity in Right-Sided Colon Cancer

**Figure 4:**
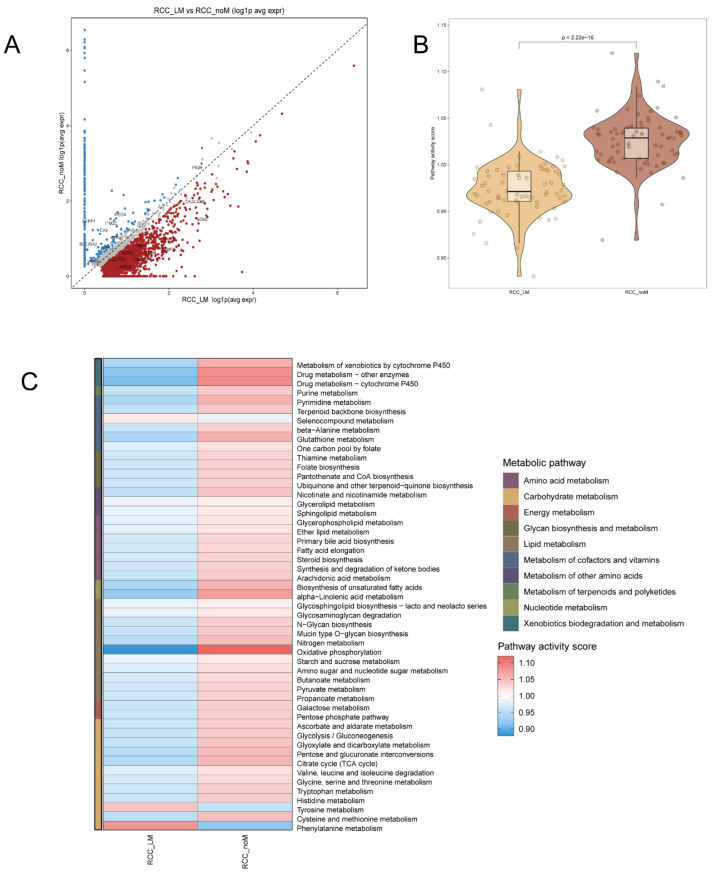
Differential gene expression and metabolic pathway activity in RCC_LM and RCC_noM tumors. (**A**) Scatter plot showing differential gene expression between the RCC_LM (liver metastasis) and RCC_noM (no metastasis) groups. Selected high-expression genes for each group are highlighted, including *LAMC2*, *ITGB4*, *COL17A1*, *AREG*, *AXIN2*, and *SOX4* for RCC_LM, and *REG4*, *PIGR*, *SEPP1*, and *LYPD8* for RCC_noM. (**B**) Violin plot comparing the Pathway activity scores between RCC_LM and RCC_noM groups, showing a significantly higher activity score for RCC_LM tumors (*p* < 0.0001). (**C**) Heatmap of metabolic pathway activity scores, highlighting key differences between RCC_LM and RCC_noM in amino acid metabolism, lipid metabolism, and nucleotide metabolism. The pathways are categorized and color-coded based on metabolic categories, with red indicating high activity and blue indicating low activity.

In our analysis of differential gene expression between the RCC_LM (liver metastasis) and RCC_noM (no metastasis) groups, we identified several genes with significantly higher expression in each group (Figure 4A). For RCC_LM, genes such as *LAMC2*, *ITGB4*, *COL17A1*, *AREG*, *AXIN2*, and *SOX4* were highly expressed, implicating these genes in epithelial–mesenchymal transition and metastasis. Conversely, RCC_noM was characterized by high expression of genes like *REG4*, *PIGR*, *SEPP1*, and *LYPD8*, suggesting a more differentiated or secretory tumor phenotype.

Further metabolic pathway analysis (Figure 4B,C) was performed using a weighted pathway activity score based on KEGG metabolism gene sets. This analysis showed significant differences in the activity of various metabolic pathways between the two groups. RCC_LM tumors exhibited higher activity in pathways related to amino acid metabolism, lipid metabolism, and nucleotide metabolism, reflecting a more aggressive and proliferative metabolic program characteristic of metastasis. In contrast, RCC_noM tumors showed lower activity in these pathways, revealing differences in metabolic reprogramming between metastatic and non-metastatic tumors.

### 3.5. Metabolic and Microenvironmental Remodeling in Metastatic and Non-Metastatic Colon Cancer

Our pathway enrichment analysis revealed distinct metabolic and microenvironmental remodeling in RCC_LM (liver metastasis) and RCC_noM (no metastasis) tumors, pointing to potential the adaptive processes that support metastatic progression versus the biosynthetic and stress-adaptive profile in non-metastatic tumors.

**Figure 5:**
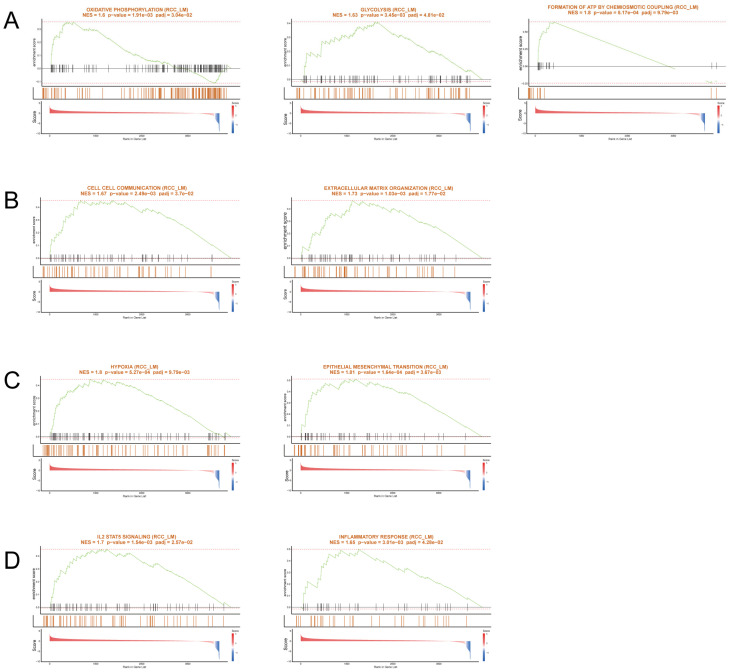
Pathway enrichment analysis of high-expression genes in RCC_LM tumors. (**A**) Gene Set Enrichment Analysis (GSEA) showing enrichment in glycolysis, oxidative phosphorylation (OXPHOS), and ATP synthesis pathways in RCC_LM, reflecting metabolic reprogramming in metastatic tumors. (**B**) Enriched pathways associated with extracellular matrix organization and cell–cell communication, suggesting ECM remodeling and cellular interaction during metastasis. (**C**) Hypoxia, apoptosis, and Epithelial–mesenchymal transition (EMT) pathways, reflecting stress-survival mechanisms and invasion in metastatic tumors. (**D**) Enrichment of IL2-STAT5 signaling and inflammatory response pathways, indicating immune activation in RCC_LM tumors.

In RCC_LM tumors, significant enrichment was observed in energy metabolism pathways, including glycolysis, oxidative phosphorylation (OXPHOS), and ATP synthesis (Figure 5A). These findings are indicative of metabolic reprogramming, with upregulated glycolysis and OXPHOS are features that confer metabolic plasticity in metastatic tumors. Additionally, the enrichment of pathways for extracellular matrix (ECM) remodeling and cell–cell communication (Figure 5B) highlights the tumor microenvironment’s reorganization to support invasion and metastasis. Epithelial–mesenchymal transition (EMT) and hypoxia (Figure 5C) pathways were also enriched, consistent with the aggressive phenotype and the shift toward a migratory, invasive tumor cell population. Inflammatory responses and IL2-STAT5 signaling (Figure 5D) further emphasize the enhanced immune activation within the metastatic context. These results underscore the dynamic remodeling of both metabolism and the tumor microenvironment that facilitates metastasis.

**Figure 6:**
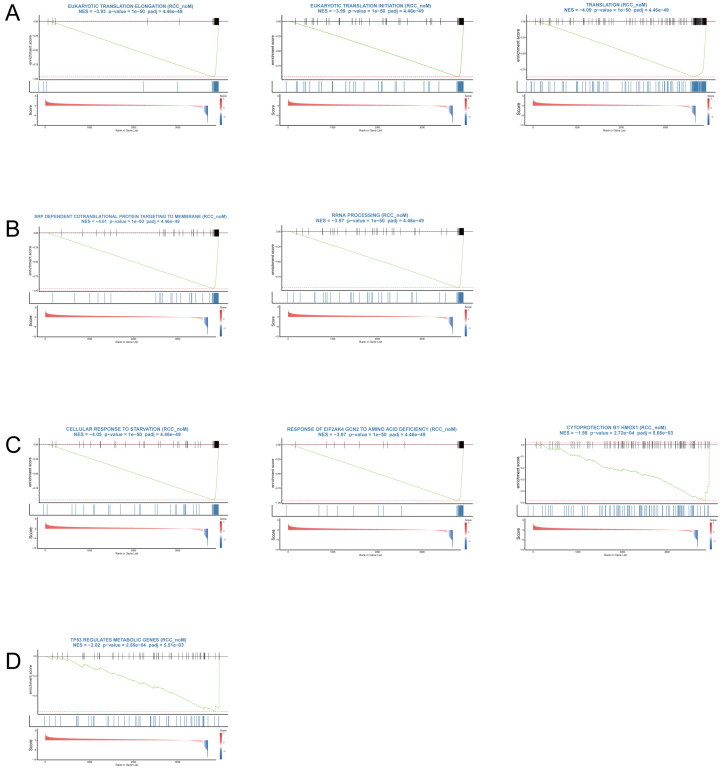
Pathway enrichment analysis of high-expression genes in RCC_noM tumors. (**A**) Gene Set Enrichment Analysis (GSEA) showing enrichment in eukaryotic translation elongation, translation initiation, and translation pathways in RCC_noM, indicating active protein synthesis in non-metastatic tumors. (**B**) Enrichment of SRP-dependent cotranslational protein targeting to membrane and rRNA processing, highlighting ribosomal activity and protein biosynthesis in RCC_noM tumors. (**C**) Enrichment of cellular response to starvation and cytoprotection by HMOX1, reflecting stress adaptation and cytoprotection in non-metastatic tumors. (**D**) TP53-regulated metabolic gene pathway enrichment, suggesting regulation of metabolism by the tumor suppressor p53 in non-metastatic tumors.

In contrast, RCC_noM tumors displayed a distinct set of enriched pathways related to translation, ribosomal function, and stress adaptation (Figure 6A,B). Key pathways such as eukaryotic translation elongation, translation initiation, and SRP-dependent cotranslational protein targeting indicated active protein synthesis and cellular maintenance in non-metastatic tumors. Additionally, pathways associated with cellular response to starvation and cytoprotection by HMOX1 (Figure 6C) suggested a stress-adaptive response to nutrient deprivation. The enrichment of the TP53-regulated metabolic gene pathway (Figure 6D) highlights the role of the tumor suppressor p53 in regulating metabolism and cellular stress response mechanisms, crucial for non-metastatic tumor survival.

Together, these findings suggest that RCC_LM tumors undergo significant metabolic and microenvironmental remodeling to promote metastatic potential, while RCC_noM tumors are characterized by a biosynthetic and stress-balancing profile, reflecting distinct adaptive programs in the context of metastatic progression.

### 3.6. Pseudotime and Differentiation Trajectories of Tumor Subclusters

**Figure 7:**
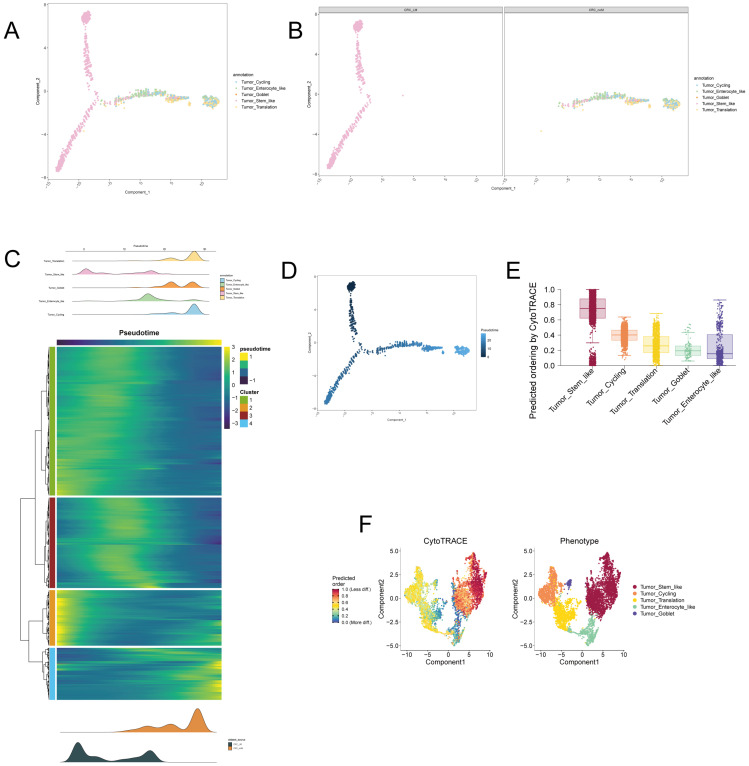
Monocle and CytoTRACE analysis of tumor subclusters. (**A**) Monocle trajectory analysis of tumor subclusters showing pseudotime progression, with Tumor Stem-like cells at the start and Tumor Translation cells at the end, suggesting a differentiation trajectory. (**B**) Monocle analysis of pseudotime ordering in RCC_LM and RCC_noM groups, showing a more stem-like phenotype in RCC_LM and a more differentiated state in RCC_noM. (**C**) Heatmap showing pseudotemporal distribution of tumor subclusters, with Tumor Stem-like cells occupying early stages and Tumor Translation and Tumor Goblet cells enriched in later stages. (**D**) CytoTRACE predicted differentiation potential across tumor subclusters, confirming the differentiation pattern seen in Monocle analysis. (**E**) Box plot showing CytoTRACE predicted pseudotemporal ordering across different tumor subclusters, with Tumor Stem-like showing the highest differentiation potential. (**F**) CytoTRACE analysis showing predicted differentiation potential for each tumor subcluster across the pseudotime trajectory, revealing a clear differentiation trend from Tumor Stem-like to Tumor Goblet subclusters.

To explore the dynamic progression and differentiation of tumor subclusters, we performed Monocle and CytoTRACE analysis to track the pseudotemporal ordering of cells and predict their differentiation potential. Both analyses revealed distinct trajectories and differentiation pathways across the tumor subclusters, shedding light on their developmental states. Monocle analysis visualized the tumor subclusters’ progression along a pseudotime continuum, with Tumor Stem-like and Tumor Translation subclusters occupying the starting and terminal points of the trajectory, respectively (Figure 7A,B,D). The Tumor Cycling and Tumor Enterocyte-like subclusters were positioned as intermediates, suggesting a transition from stem-like to more differentiated states (Figure 7A,B,D). In the RCC_LM (liver metastasis) group, the Tumor Stem-like subcluster predominated, revealing an enrichment of that metastatic tumors are enriched in stem-like phenotypes, while RCC_noM (no metastasis) tumors displayed a more differentiated pseudotime profile (Figure 7C). The heatmap further supported these findings, showing that Tumor Stem-like cells are predominantly at the early pseudotime stages, while Tumor Translation and Tumor Goblet subclusters are enriched in the later stages, supporting differentiation from stem-like populations (Figure 7C). CytoTRACE analysis predicted that Tumor Stem-like cells have the highest differentiation potential, aligning with the pseudotime progression observed in Monocle (Figure 7E,F). The predicted pseudotemporal ordering and differentiation potential across the subclusters demonstrated that Tumor Stem-like and Tumor Cycling subclusters dominate the early stages, while Tumor Translation, Tumor Enterocyte-like, and Tumor Goblet subclusters represent more differentiated tumor populations (Figure 7E,F). These findings underscore the complex trajectories and differentiation pathways within the tumor microenvironment.

## 4. Discussion

This study systematically analyzed the cellular heterogeneity of right-sided colon cancer primary tumors to uncover the features defining their liver metastatic potential using single-cell RNA sequencing technology. Our results reveal significant differences between metastatic and non-metastatic right-sided colon cancers at multiple levels, including cellular composition, genomic alterations, metabolic reprogramming, and cellular differentiation trajectories, providing important single-cell level evidence for understanding the molecular mechanisms of colorectal cancer liver metastasis.

Our study found that in the RCC_LM group, the proportion of tumor stem-like subpopulations was significantly higher, with these cells highly expressing stemness-related genes such as *AXIN2*, *ASCL2*, and *KLF5*. This finding aligns with previous research demonstrating the critical role of tumor stem cells in metastasis [32,33,34]. Tumor stem cells possess self-renewal capacity and multi-directional differentiation potential, which enable them to survive in the circulatory system and colonize distant organs [35,36]. Monocle and CytoTRACE analyses further confirmed that tumor stem-like cells are located at the starting point of the differentiation trajectory, exhibiting the highest differentiation potential, suggesting that metastatic tumors retain greater cellular plasticity.

In contrast, the RCC_noM group was enriched with more enterocyte-like subpopulations, expressing differentiation markers such as *GUCA2A/B*, *ALDOB*, and *KRT20*, consistent with the fact that non-metastatic tumors have a more mature differentiation phenotype. This difference in differentiation status may reflect the biological characteristic that tumor cells need to dedifferentiate in order to acquire migration and invasion abilities during metastasis. Notably, epithelial–mesenchymal transition (EMT) pathways were significantly enriched in the RCC_LM group, further supporting the close connection between stem-like phenotypes and metastatic capacity.

CNV analysis revealed that tumor cells in the RCC_LM group exhibited significantly higher copy number variation (CNV) scores, demonstrating more severe genomic instability in metastatic tumors. Genomic instability is considered a major driver of tumor evolution and adaptive evolution, as it generates genetic diversity that provides the raw material for tumor cells to adapt to selective pressures [37,38]. During metastasis, tumor cells need to overcome multiple barriers, including detachment from the primary site, survival in the circulatory system, extravasation to target organs, and proliferation in a heterogeneous microenvironment [39,40]. Increased genomic instability may lead to the emergence of clones with metastatic advantages, thus promoting liver metastasis.

Additionally, we observed an increase in the proportion of cells in the G1 phase in the RCC_LM group, indicating altered cell cycle dynamics that may be associated with a dormant or migratory phenotype rather than enhanced proliferation. Cell cycle dysregulation and genomic instability often interact to promote tumor malignancy [41,42,43]. These observations highlight that therapeutic strategies targeting cell cycle checkpoints and DNA damage repair pathways may hold potential value for the prevention or treatment of colorectal cancer liver metastasis.

Our metabolic pathway analysis revealed significant metabolic differences between the RCC_LM and RCC_noM groups. RCC_LM tumors exhibited synergistic upregulation of glycolysis, oxidative phosphorylation, and ATP synthesis pathways, a metabolic plasticity known as the “metabolic hybrid phenotype,” which allows tumor cells to flexibly adjust energy production according to the microenvironment [44]. Under hypoxic or nutrient-deprived conditions, tumor cells rely on glycolysis for rapid ATP production, whereas in the presence of oxygen and nutrients, they utilize oxidative phosphorylation for higher energy efficiency [45]. This metabolic flexibility—reflected by the co-activation of glycolysis and oxidative phosphorylation in RCC_LM tumors—may enable tumor cells to adapt to fluctuating oxygen and nutrient availability in the bloodstream and distant organs, a key feature of metastatic progression [46].

RCC_LM tumors also showed increased activity in amino acid metabolism, lipid metabolism, and nucleotide metabolism pathways. Amino acids not only serve as building blocks for protein synthesis but also regulate various signaling pathways; lipid metabolism provides materials for cell membrane synthesis and participates in signal transduction; enhanced nucleotide metabolism supports DNA and RNA synthesis required for rapid proliferation [47,48]. These metabolic changes collectively form a metabolic network that supports the invasive growth of metastatic tumors. This metabolic rewiring and altered cell cycle progression are consistent with the activation of oncogenic signaling cascades such as the PI3K/AKT/mTOR pathway, which prior experimental studies have shown to centrally regulate colorectal cancer cell metabolism, proliferation, and metastasis [49,50].

In contrast, RCC_noM tumors were enriched in translation initiation, elongation, and ribosomal-related pathways, reflecting high levels of protein synthesis activity. We also observed enrichment of cellular responses to starvation and HMOX1-mediated cytoprotection pathways, as well as activation of TP53-regulated metabolic gene pathways, indicating that non-metastatic tumors are in a stress-adaptive state. This metabolic pattern may reflect non-metastatic tumors’ attempts to maintain cellular homeostasis and resist stress, rather than actively engaging in metastatic spread [51].

RCC_LM tumors exhibited significant enrichment of pathways related to extracellular matrix (ECM) remodeling and cell–cell communication, indicating active crosstalk between malignant cells and the tumor microenvironment (TME). This ECM reprogramming, driven by genes such as *LAMC2*, *ITGB4*, and *COL17A1*, likely facilitates stromal invasion and creates a permissive niche for metastatic dissemination. ECM not only provides physical support for tumor cells but also regulates cell behavior through integrin signaling [52]. We found that *LAMC2*, *ITGB4*, and *COL17A1* were highly expressed in RCC_LM, genes encoding laminin, integrins, and collagen, which are directly involved in ECM composition and remodeling. ECM restructuring creates favorable conditions for tumor cell invasion and migration, a critical step in the metastatic cascade [53].

Interestingly, we observed the enrichment of inflammatory response and IL2-STAT5 signaling pathways in RCC_LM, indicating more active immune activation within the metastatic tumor microenvironment. These findings may reflect underlying dynamics in T-cell differentiation and memory programming, which recent studies have shown to be critical for sustaining anti-tumor immunity and shaping the immune microenvironment [54,55]. This finding may seem paradoxical, as the immune system should theoretically suppress tumor metastasis. However, increasing evidence suggests that chronic inflammation can promote tumor progression, and certain immune cell subpopulations may even support tumor invasion and metastasis [56,57]. Moreover, the enrichment of hypoxia pathways in RCC_LM may induce the formation of an immune-suppressive microenvironment, allowing tumor cells to escape immune surveillance. These findings emphasize the complexity of tumor-immune interactions in liver metastasis, warranting further investigation into specific immune cell subpopulations and molecular mechanisms [58].

Through Monocle and CytoTRACE analyses, we reconstructed the differentiation trajectories of tumor cells, revealing a continuous transition from stem-like states to various differentiated endpoints. This trajectory analysis not only confirmed the hierarchical organization within the tumor but also highlighted the important role of cellular plasticity in metastasis [59,60]. RCC_LM tumors were enriched in early stages of the differentiation trajectory, while RCC_noM tumors leaned toward a more differentiated state, consistent with our findings in cellular subpopulation proportions.

Cellular plasticity allows tumor cells to transition between different differentiation states, a crucial ability during metastasis [61]. Tumor cells may need to undergo EMT to acquire migration ability and later undergo mesenchymal–epithelial transition (MET) at the metastatic site to re-establish an epithelial phenotype [62]. Our data indicate that metastatic tumors retain greater cellular plasticity, enabling them to adapt to different stages of the metastatic cascade.

The metastatic-related features identified in this study have important clinical translational potential. First, the proportion of tumor stem-like subpopulations, CNV scores, and the expression of specific genes (such as *LAMC2*, *ITGB4*, and *AXIN2*) may serve as biomarkers for predicting the risk of liver metastasis, helping identify high-risk patients and guide adjuvant therapy decisions. Second, the metabolic vulnerabilities and pathway dependencies we identified provide potential therapeutic targets. For instance, metabolic inhibitors targeting glycolysis or oxidative phosphorylation, or drugs targeting EMT, ECM remodeling, and stem cell signaling pathways, may help prevent or treat liver metastasis.

Beyond their translational potential, our findings offer a broader biological insight into the ecosystem driving metastasis. Collectively, they underscore the importance of the tumor microenvironment in shaping metastatic behavior in right-sided colon cancer. The observed enrichment of ECM-related pathways, immune activation signatures, and metabolic reprogramming in RCC_LM tumors converges to suggest a coordinated remodeling of both cancer cells and their surrounding stroma. These interactions may create a permissive ecosystem that supports tumor cell survival, immune evasion, and eventual liver colonization. Future studies integrating spatial transcriptomics and functional assays will be essential to dissect the cellular and molecular architecture of this metastatic niche.

Moreover, our study highlights the value of single-cell technology in tumor heterogeneity research. Traditional bulk tissue sequencing obscures the diversity of tumor cell populations and fails to resolve the distinct stromal and immune components of the tumor microenvironment, while single-cell analysis allows for precise identification of key metastatic-driving subpopulations and their characteristic molecular programs. This high-resolution analysis method offers unprecedented insights into cancer biology.

## 5. Study Limitations

There are several limitations in this study that should be considered. First, the relatively small sample size (8 cases) may limit the statistical power and generalizability of the results. Although single-cell sequencing provides thousands of cells per patient, inter-patient heterogeneity may not have been fully captured. Second, this study relied on retrospective analysis of public datasets and lacked clinical follow-up data, preventing the assessment of the correlation between the identified biomarkers and patient prognosis. Third, our analysis was primarily based on transcriptomic data, lacking protein-level validation and mechanistic studies. Future work should validate key findings in independent cohorts and use in vitro and in vivo experiments to elucidate the specific molecular mechanisms. Furthermore, a critical limitation of our study is that the samples with liver metastasis (RCC_LM) and those without metastasis (RCC_noM) were derived from two distinct public datasets (GSE245552 and GSE188711). In addition to the technical considerations, the clinical annotation of the samples presents inherent limitations. First, the RCC_LM group is specifically defined by the presence of synchronous liver metastasis. Whether these findings can be generalized to patients who develop metachronous metastasis (i.e., metastasis occurring months or years after primary tumor resection) remains unknown, as the biological mechanisms driving early simultaneous dissemination may differ from those governing late recurrence. Second, the available clinical metadata from the public datasets is limited. Detailed information on tumor stage (TNM), microsatellite instability (MSI) status, patient age, sex, and long-term follow-up data were not uniformly available for all samples. This precludes a more nuanced analysis of how these factors might interact with the cellular programs we identified. Future studies with prospectively collected, uniformly processed cohorts that include comprehensive clinical annotation are essential to validate and extend our findings. Although we employed the Harmony algorithm to rigorously correct for potential batch effects arising from different data sources prior to all downstream analyses, it remains possible that residual technical or biological confounding factors inherent to the original studies could influence our results. While our integration strategy aims to disentangle true biological signals associated with metastatic potential from dataset-specific artifacts, this inherent data structure represents a significant caveat that must be considered when interpreting our findings. Future validation in a prospectively collected, uniformly processed cohort is essential.

Additionally, this study only analyzed primary tumor samples and did not include paired metastatic lesions or normal tissue. Comparing primary tumors and metastases will provide a more comprehensive understanding of cellular evolution during metastasis. Longitudinal samples (before and after treatment, during disease progression) will provide a dynamic view of tumor evolution. And We acknowledge that the Wilcoxon rank-sum test used for differential expression analysis is a cell-level test. While this is a standard and robust method in single-cell analysis, future studies could employ mixed-effects models or other advanced statistical methods that account for per-patient variability to further refine the findings. Finally, although we identified several metastatic-related pathways, the interactions and hierarchical relationships between these pathways remain unclear, warranting system biology methods for integrated analysis.

## 6. Conclusions

This study provides a single-cell analysis of right-sided colon cancer, revealing multi-dimensional molecular features associated with liver metastasis. We found that metastatic tumors are enriched in tumor stem-like cells, exhibiting higher genomic instability, metabolic plasticity, and microenvironment remodeling capabilities. These findings deepen our understanding of the mechanisms of colorectal cancer liver metastasis and provide a theoretical basis for developing new predictive biomarkers and therapeutic strategies. As single-cell technologies continue to develop and clinical translational research progresses, these findings hold the potential to ultimately improve clinical outcomes for metastatic colorectal cancer patients.

## Data Availability

The data used in this study are available from the corresponding authors upon request.

## Supplementary Materials

The following supporting information can be downloaded at: https://www.mdpi.com/article/10.3390/biomedicines14030563/s1, Figure S1: Additional gene expression patterns in the right-sided colon cancer tumor microenvironment. Figure S2. Harmony effectively corrects for batch effects between the two source datasets.
