# Supplementary material for: Single-Cell Transcriptomic Landscape of Right-Sided Colon Cancer Reveals Cellular and Molecular Features of Metastatic Potential
Source: Biomedicines. 2026 Feb 28;14(3):563. doi: 10.3390/biomedicines14030563 (PMC13024220; doi:10.3390/biomedicines14030563)
Supplement: Supplementary file 1 [file biomedicines-14-00563-s001.zip › biomedicines-4116386-supplementary.pdf]

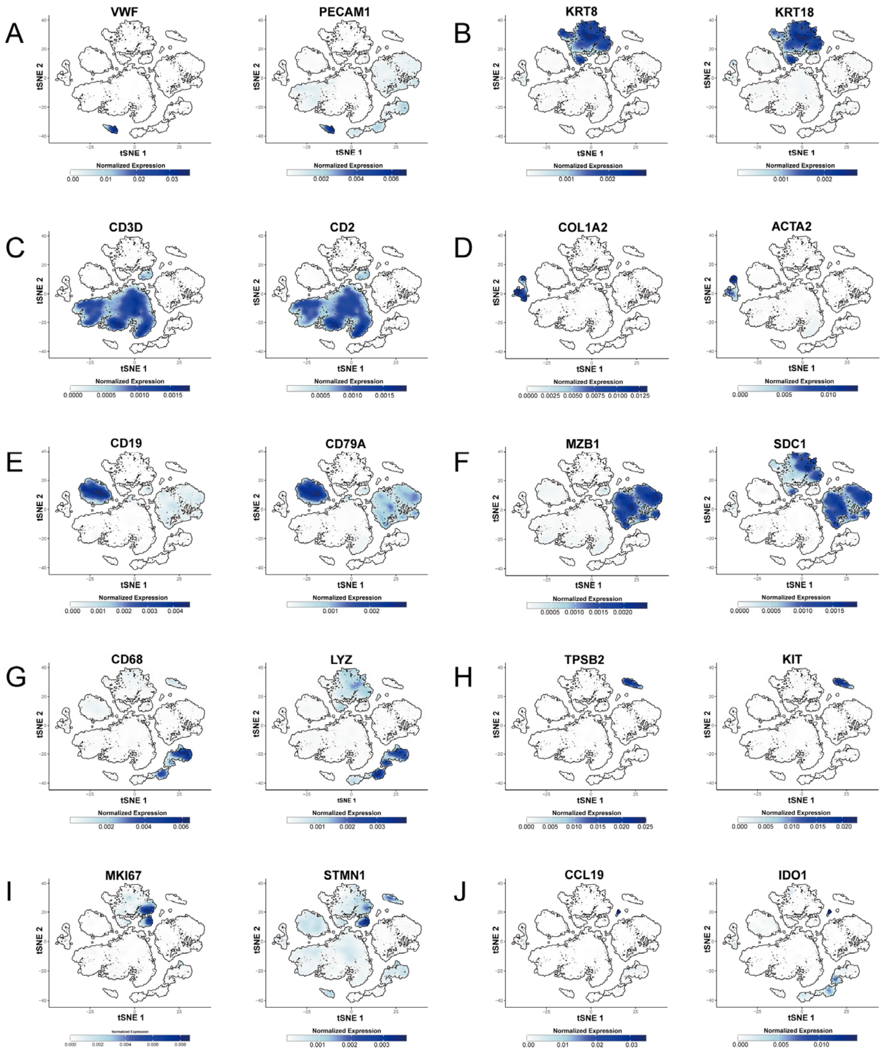

Figure S1. Additional gene expression patterns in the right-sided colon cancer tumor microenvironment. (A-J) t-SNE plots showing the expression of key marker genes across different cell clusters. For each cluster, two representative marker genes are shown; the placement of genes in left versus right subpanels is arbitrary and does not convey biological ordering or priority. Expression intensity is color-coded from low (blue) to high (dark blue), providing further insights into the heterogeneity of the tumor microenvironment and the identity of the different cell populations.

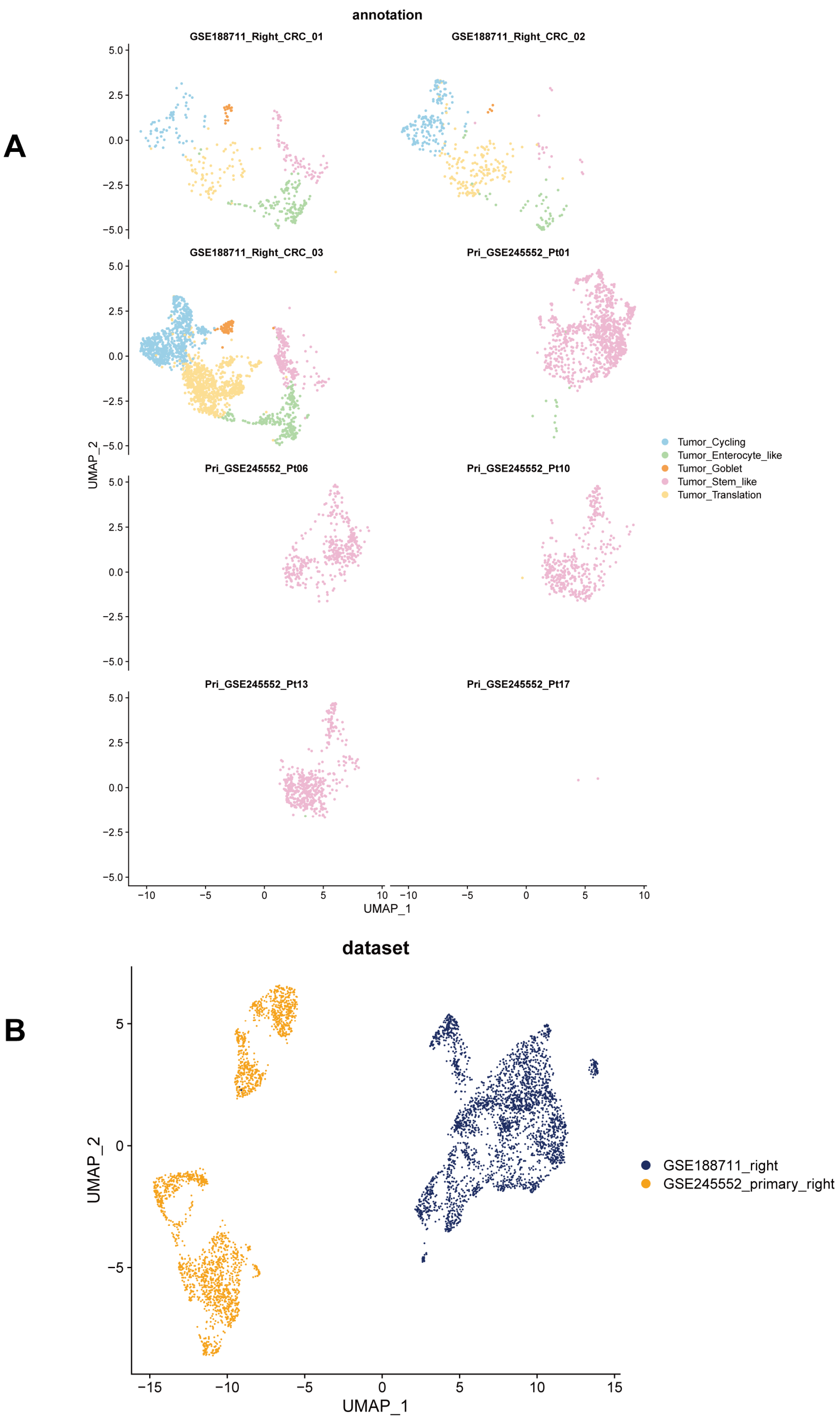

Figure S2. Harmony effectively corrects for batch effects between the two source datasets. (A) UMAP embedding of tumor cells after Harmony integration, colored by annotated cell subtypes and faceted by individual sample. Cells from different samples cluster primarily by biological identity rather than dataset origin, indicating successful removal of technical batch effects. (B) UMAP embedding of the same tumor cells before Harmony correction, colored by dataset source: orange = GSE188711, blue = GSE245552. In the absence of batch correction, cells form two distinct, non-overlapping clusters strictly segregated by dataset, demonstrating a strong underlying batch effect.
